# Adjuvantation of Influenza Vaccines to Induce Cross-Protective Immunity

**DOI:** 10.3390/vaccines9020075

**Published:** 2021-01-21

**Authors:** Zhuofan Li, Yiwen Zhao, Yibo Li, Xinyuan Chen

**Affiliations:** Biomedical & Pharmaceutical Sciences, College of Pharmacy, University of Rhode Island, 7 Greenhouse Road, Avedisian Hall, Room 480, Kingston, RI 02881, USA; zhuofan_li@uri.edu (Z.L.); yiwen_zhao@uri.edu (Y.Z.); yibo_li@uri.edu (Y.L.)

**Keywords:** vaccine adjuvant, influenza vaccine, cross-protection, MF59, AS03, physical adjuvant

## Abstract

Influenza poses a huge threat to global public health. Influenza vaccines are the most effective and cost-effective means to control influenza. Current influenza vaccines mainly induce neutralizing antibodies against highly variable globular head of hemagglutinin and lack cross-protection. Vaccine adjuvants have been approved to enhance seasonal influenza vaccine efficacy in the elderly and spare influenza vaccine doses. Clinical studies found that MF59 and AS03-adjuvanted influenza vaccines could induce cross-protective immunity against non-vaccine viral strains. In addition to MF59 and AS03 adjuvants, experimental adjuvants, such as Toll-like receptor agonists, saponin-based adjuvants, cholera toxin and heat-labile enterotoxin-based mucosal adjuvants, and physical adjuvants, are also able to broaden influenza vaccine-induced immune responses against non-vaccine strains. This review focuses on introducing the various types of adjuvants capable of assisting current influenza vaccines to induce cross-protective immunity in preclinical and clinical studies. Mechanisms of licensed MF59 and AS03 adjuvants to induce cross-protective immunity are also introduced. Vaccine adjuvants hold a great promise to adjuvant influenza vaccines to induce cross-protective immunity.

## 1. Introduction

Influenza is a highly contagious viral infectious disease [1]. Based on the World Health Organization (WHO), influenza causes 3–5 million severe illnesses and 290,000–650,000 deaths each year worldwide. Influenza viruses are negative-sensed, single-stranded, segmented RNA viruses [2,3]. Influenza viruses belong to the virus family Orthomyxoviridae [2,3]. There are four types of influenza viruses: A, B, C, and D [4]. Human influenza is often caused by type A and B viruses [3]. Hemagglutinin (HA) and neuraminidase (NA) are highly abundant viral surface proteins with important roles in viral infection and release [3]. There are a total of 18 HA and 11 NA subtypes, giving rise to 198 possible influenza A subtypes [3]. Not all influenza A subtypes have been found in nature [4]. The natural hosts of influenza A viruses are wild aquatic birds, although influenza A viruses have also adapted to a variety of avian and mammalian species [5]. H1N1 and H3N2 are two major influenza A subtypes that cause annual influenza epidemics in humans [4]. Different from influenza A viruses, influenza B viruses have only two lineages (Victoria and Yamagata) and only infect humans and seals [3,6]. Influenza viruses have a high mutation rate due to the lack of proofreading of RNA-dependent RNA polymerase [7]. Based on reports, the annual mutation rates for the non-structural genes of influenza A and B viruses are about 2.0 × 10^−3^ and 0.6 × 10^−3^ nucleotide substitutions/site, respectively [8]. The high mutation rates of influenza A viruses cause frequent sequence changes of HA, leading to antigenic drift [3,9,10]. Antigenic drift enables influenza viruses to evade host immune surveillance and cause epidemics [3,9]. The co-infection of different influenza A subtypes can also cause reassortment, a process by which influenza viruses exchange gene segments and generate new viruses [9]. Due to the lack of preexisting immunity, the reassortant viruses have a high risk to cause influenza pandemics if sufficient human-to-human transmission can be established [9,11,12]. Four major influenza pandemics have occurred in human history: the 1918 H1N1 pandemic, the 1957 H2N2 pandemic, the 1968 H3N2 pandemic, and the 2009 H1N1 pandemic [13,14]. The 1918 influenza pandemic infected about one-third of the global population and claimed 50 million lives [15]. The 1957 H2N2 and 1968 H3N2 influenza pandemics each claimed about one million lives [13,16]. The most recent 2009 influenza pandemic claimed an estimated 151,700−575,400 lives [16,17]. The 1957 H2N2 influenza contains three genes from an avian influenza A virus, and the 1968 H3N2 influenza contains two genes from an avian influenza A virus [13], while the 2009 H1N1 influenza contains a unique combination of genes from avian, human, and swine influenza A viruses [18].

Influenza vaccines remain the most effective and cost-effective means to control influenza [19,20]. Yet, current influenza vaccines mainly induce strain-specific protection with little or no protection against drifted or shifted viruses. Universal influenza vaccines are under active development to induce broad cross-protective immunity [21,22,23]. In addition to a universal influenza vaccine approach, the incorporation of vaccine adjuvants has been found to broaden influenza vaccine-induced immune responses. This review focuses on introducing vaccine adjuvants that are capable of assisting current influenza vaccines to induce broad cross-protective immunity.

## 2. Current Influenza Vaccines and Limitations

Influenza vaccines are produced each year to mitigate influenza-caused morbidity and mortality [19,20]. Different types of influenza vaccines are available in the global market (Table 1). Based on the number of vaccine viral strains, influenza vaccines can be divided into trivalent or quadrivalent vaccines with the former containing two type A (H1N1, H3N2) and one type B (Victoria or Yamagata) strains and the latter containing two type A (H1N1, H3N2) and two type B (Victoria and Yamagata) strains [24,25]. Influenza vaccines can also be classified into inactivated or attenuated vaccines [26]. Inactivated vaccines can be further divided into subunit, split-virion, and virosome vaccines [26]. Live-attenuated influenza vaccines undergo limited proliferation in respiratory epithelial cells following intranasal (IN) delivery [26]. Inactivated subunit vaccines are prepared by the disruption of viruses by detergents followed by purification of HA and NA as the major vaccine antigens [26]. Inactivated split-virion vaccines are prepared by the disruption of viruses by detergents without further purification [26]. As a result, split-virion vaccines contain additional viral components as compared to subunit vaccines [26]. Virosome vaccines are prepared by the detergent solubilization of viral membrane followed by nucleocapsid removal and then detergent removal to allow the viral membrane to self-assemble into virus-like particles (VLPs) [27]. Influenza virosome vaccines simulate native viruses in cell binding and membrane fusion [27]. Influenza virosome vaccine (Inflexal V) is the only influenza vaccine that has been approved for all age groups [28]. Based on the production methods, influenza vaccines can be classified into egg, cell, or recombinant protein-based [26,29]. Embryonic eggs have a long history of being used to expand influenza viruses to produce influenza vaccines [30]. Yet, embryonic egg-based influenza vaccine manufacturing is a lengthy process and also depends on egg supply [29]. To overcome these limitations, cell-based influenza vaccine manufacturing has been developed, in which influenza viruses are propagated in mammalian cells, such as Madin–Darby Canine Kidney (MDCK) cells [31]. Cell-based influenza vaccine manufacturing is not limited by egg supply and less likely to introduce viral mutations [26]. Recombinant influenza vaccines have been developed based on recombinant DNA technologies [32]. One such vaccine (Flublok) is produced in insect cells using Baculovirus Expression Systems [33]. As compared to egg and cell-based vaccines, recombinant influenza vaccines can be more quickly produced without the use of live viruses [29]. Based on the delivery routes, influenza vaccines can be classified into intramuscular (IM), intradermal (ID), and IN vaccines [19,34]. Most of the inactivated influenza vaccines are delivered into the muscular tissue by hypodermic needles, while live-attenuated vaccines are delivered by Intranasal Sprayer to the upper respiratory tract [34]. IN influenza vaccination provides an important alternative to needle-based IM vaccination for those who have needle phobia [35]. Needle-free Jet Injectors, such as PharmaJet and Bioject ZetaJet, have been also developed for the IM delivery of inactivated influenza vaccines [36,37,38]. Due to the richness of antigen-presenting cells (APCs) in the skin [39,40], inactivated influenza vaccines are also approved for ID delivery by Intradermal Microinjection Systems and MicronJet600 to save vaccine dose and cost [41,42]. Based on the usage, influenza vaccines can be divided into seasonal, pre-pandemic, and pandemic vaccines [32]. Seasonal influenza vaccines are made based on viral strains predicted to circulate in the next flu season [20,43]. Pre-pandemic influenza vaccines are prepared based on viral strains with a high risk to cause influenza pandemics, such as highly pathogenic avian influenza (HPAI) H5N1 and H7N9 viruses [44,45]. Pre-pandemic influenza vaccines are stockpiled to facilitate a quick response in case of real pandemics [44]. Pandemic influenza vaccines are made based on pandemic viral stains to control the spread of pandemic influenza [46].

Current influenza vaccines mainly induce neutralizing antibodies against the globular head of HA, lacking cross-protection against drifted or shifted viral strains [21,26,29]. HA is a type I transmembrane glycoprotein and forms trimeric spikes on the influenza viral surface [47,48,49]. To form functional HA, HA precursor HA0 needs to be cleaved by cellular protease to form HA1 and HA2 linked by disulfide bonds [50]. Functional HA is composed of a globular head and a stem domain [47]. The globular head is comprised of part of HA1, and the stem domain is comprised of the remaining HA1 and part of HA2 [47]. The remaining HA2 is composed of a transmembrane domain and a cytosolic tail [47]. HA plays crucial roles in viral infection. HA binds to sialic acids on the host cell surface and mediates the fusion of viral envelop with the late endosomal membrane of host cells^2^. Based on the sequence and antigenic relatedness of HA, influenza A viruses can be divided into group 1 (H1, H2, H5, H6, H8, H9, H11, H12, H13, H16, H17, and H18) and group 2 viruses (H3, H4, H7, H10, H14, and H15) [47].

Due to the immune selection pressure, the globular head of HA undergoes constant antigenic drift [10,47], which requires influenza vaccines to be manufactured each year. Considering that influenza viruses to circulate in the next flu season are predicted based on surveillance [20,43], the selected vaccine viral strains may not exactly match circulating viral strains. Vaccine mismatch often leads to low vaccine effectiveness and surging influenza cases [20,43]. In the 2014–2015 flu season, influenza A H3N2 viruses drifted significantly after the selection of vaccine viral strains [51]. Consequently, influenza vaccine effectiveness against the drifted H3N2 strain was only 6%, and the overall vaccine effectiveness was only 19% [52]. There were also significant vaccine mismatches in the 2004–2005 and 2005–2006 flu seasons, which resulted in low vaccine effectiveness in the two flu seasons (10 and 21%, respectively) [53]. In contrast, vaccine effectiveness is usually between 40 and 60% with a good vaccine match [52]. Egg-based vaccine manufacturing sometimes introduces mutations in immunodominant regions of HA, leading to reduced vaccine effectiveness [26].

Due to the high risk of HPAI H5N1 and H7N9 viruses to cause influenza pandemics, pre-pandemic H5N1 and H7N9 vaccines have been stockpiled [44,54]. HPAI H5N1 viruses mainly infect birds and are highly contagious among them [11]. The first human infection of H5N1 was identified in 1997 amid a poultry outbreak in Hong Kong, China [45]. H5N1 viruses have infected about 700 people in primarily 15 countries in Asia, Africa, the Pacific, Europe and the Near East with ≈60% lethality rate [45]. The first human infection of H7N9 occurred in China in 2013 [45]. Ever since, H7N9 has caused almost annual epidemics in humans [55]. The largest epidemic caused about 766 human infections in 2016–2017 [55]. Overall, H7N9 caused more than 1500 human infections with a lethality rate of ≈39% [55]. Although most H5N1 and H7N9 infections in humans were due to the close contact with poultry, human-to-human transmission may occur as a result of viral gene reassortment [45]. Different types of H5N1 and H7N9 vaccines have been developed and evaluated in clinical studies that include subunit, split-virion, and whole-inactivated vaccines [54,56]. H5N1 and H7N9 vaccines often require two immunizations and high antigen amounts (45 or 90 µg) to induce protective immune responses due to their low immunogenicity [57,58,59,60]. Molecular epidemiology studies found that H5N1 viruses had evolved into different clades and subclades, and H7N9 viruses had evolved into distinct lineages [61,62]. Similar to the seasonal influenza vaccine, H5N1 and H7N9 vaccines alone lack the ability to induce cross-clade or cross-lineage immunity [59,63].

## 3. Vaccine Adjuvants

Adjuvants(Latin word “adjuvare”, meaning to “help”) are traditionally defined as chemicals added to vaccine preparations to help vaccines to work better [64,65]. Considering vaccines are mainly given to healthy populations, adjuvants need to have a high level of safety to be approved for human use [64,66,67]. Experimental adjuvants, such as complete and incomplete Freund’s adjuvants, lipopolysaccharide (LPS), and a variety of cytokines, possess potent vaccine adjuvant effects and yet are less likely to be approved for human use due to their high risk to induce significant local or systemic adverse reactions [64,68,69].

Aluminum salt-based Alum adjuvant represents the first and most widely used adjuvant in the globe [70,71,72]. Alum adjuvant was discovered in 1920s by Raman and Glenny based on the observation that the precipitation of vaccines on insoluble aluminum salts could enhance vaccine-induced immune responses [70,71,72]. Then, Alum adjuvant was incorporated into tetanus and diphtheria vaccines and broadly used in humans [70,71,72]. Interestingly, Alum adjuvant was ineffective to enhance seasonal, pre-pandemic, or pandemic influenza vaccine efficacy [73,74,75,76]. Alum adjuvant mainly induces type 2 T helper (Th2)-biased immune responses and was originally thought to act through an “antigen depot”. In 2012, Hutchison et al. found that the removal of Alum depot as early as 2 h after administration had no significant impact on antigen-specific T and B cell responses [77]. Instead, Kool et al. found that Alum adjuvant could induce uric acid release and activate inflammatory dendritic cells (DCs) to mediate its adjuvant effects [78]. Several studies also found that Alum adjuvant could activate the Nod-like receptor (NLR) family pyrin domain containing 3 (NALP3) inflammasome, leading to Caspase-1 activation and interleukin 1β (IL-1β) release [79,80,81]. Yet, the depletion of NALP3 only affected antigen-specific IgE but not IgG1 or IgG2 antibody production [81,82,83]. More work is needed to elucidate how Th2-biased immune responses are induced by Alum-adjuvanted vaccines [83,84]. Alum adjuvant remained the only adjuvant for more than half a century until squalene emulsion-based MF59 adjuvant was approved by European Authorities in 1997 to enhance seasonal influenza vaccine efficacy in the elderly [85,86,87]. MF59 was approved in 2015 to enhance seasonal influenza vaccine efficacy in the elderly in the United States [88,89]. MF59 has been also licensed to boost seasonal influenza vaccine efficacy in children aged 6 months to 2 years old in Canada since 2015 [90]. In addition to seasonal influenza vaccines, MF59 has been also incorporated into pre-pandemic H5N1 and H7N9 vaccines to spare vaccine doses [91,92]. Overall, millions of MF59-containing influenza vaccine doses have been administered in humans with a good safety profile [85,86,87]. After MF59, several adjuvant system (AS)-based adjuvants, such as AS01, AS03, and AS04, were approved for human use [93,94]. AS04 is prepared by the adsorption of monophosphoryl lipid A (MPL) onto Alum adjuvant and was approved to improve human papillomavirus (HPV) vaccine efficacy in 2009 [95,96]. MPL is a non-toxic derivative of LPS and a potent Toll-like receptor (TLR) 4 agonist [66,97]. AS03 represents another squalene emulsion-based adjuvant [98,99]. AS03 was incorporated into the influenza pandemic 2009 H1N1 vaccine in Europe [100,101]. AS03 was also incorporated into the pre-pandemic H5N1 vaccine stockpiled in the United States [102]. AS01 and AS02 are MPL and QS21-containing liposome and water-in-oil emulsion, respectively [93,103,104]. AS01 was approved to boost pre-erythrocytic RTS, S malaria vaccine efficacy in 2015 [94,105,106]. Recently, CpG 1018 was approved to boost hepatitis B VLP vaccine efficacy [107,108]. CpG 1018 is a 22-mer oligonucleotide and activates TLR9 [107,109]. An overview of currently approved vaccine adjuvants is summarized in Table 2.

Adjuvant development lagged behind vaccine development for a long time. This trend is gradually changing with increasing understanding of adjuvant mechanisms and more investments in adjuvant research and development. The various benefits of adjuvants include enhancing vaccine-induced immune responses, improving vaccine efficacy in the elderly, sparing vaccine doses, inducing early-onset and long-lasting immune responses, and eliciting cross-protective immunity [64,66]. Different types of adjuvants have been explored to increase seasonal, pre-pandemic, and pandemic influenza vaccine efficacy, spare vaccine doses, and broaden vaccine-induced immune responses. This review focuses on introducing vaccine adjuvants capable of assisting current influenza vaccines to induce cross-protective immunity.

## 4. Vaccine Adjuvants Assist Influenza Vaccines to Induce Cross-Protective Immunity

Licensed adjuvants, such as MF59 and AS03, are incorporated into seasonal, pre-pandemic, and pandemic influenza vaccines to enhance vaccine efficacy in the elderly or spare vaccine doses. MF59 and AS03-adjuvanted influenza vaccines were found to induce cross-protective immunity in humans (Table 3). In addition to licensed adjuvants, various types of experimental adjuvants were found to also assist influenza vaccines to induce cross-protective immunity, such as TLR agonists (e.g., MPL, Imiquimod, CpG), saponin-containing adjuvants, and non-traditional physical adjuvants (Table 4).

### 4.1. MF59

#### 4.1.1. Induction of Cross-Protective Immunity against Seasonal Influenza Vaccine

Minutello et al. conducted a small-scale study to compare the relative immunogenicity of MF59-adjuvanted seasonal influenza vaccine with non-adjuvanted vaccine in elderly ambulatory patients in the 1992–1995 flu seasons [110]. MF59-adjuvanted vaccine induced higher hemagglutination inhibition (HI) geometric mean titer (GMT) and higher seroconversion rates than the non-adjuvanted vaccine in each flu season [110]. Interestingly, the MF59-adjuvanted but not the non-adjuvanted vaccine in the 1992–1993 flu season induced significant HI GMT against heterologous viruses used to produce seasonal influenza vaccines for the 1993–1994 flu season [110]. This study hinted that MF59 might broaden influenza vaccine-induced antibody responses against non-vaccine viral strains. This hypothesis was confirmed in a later study, in which Ansaldi et al. compared HI GMT and seroconversion rates of MF59-adjuvanted and non-adjuvanted vaccines in the 2004–2005 flu season against heterologous H3N2 viruses circulated in four prior and two subsequent flu seasons in the elderly [111]. The MF59-adjuvanted vaccine induced significantly higher HI GMT against heterologous H3N2 strains circulated in subsequent flu seasons and significantly higher seroconversion rates against heterologous H3N2 strains circulated in prior and subsequent flu seasons as compared to non-adjuvanted vaccine [111]. In addition to the elderly, MF59-adjuvanted seasonal influenza vaccine also induced heterologous immunity in young children. Nolan et al. found that the MF59-adjuvanted seasonal influenza vaccine induced significantly higher HI GMT and seroconversion rates against heterologous type A and B viruses in infants and young children than the non-adjuvanted vaccine [112].

#### 4.1.2. Induction of Cross-Clade Immunity against Pre-Pandemic Influenza Vaccine

MF59 adjuvant was explored to spare pre-pandemic H5N1 vaccine doses and induce cross-clade protection. Banzhoff et al. found that two priming doses of MF59-adjuvanted H5N1 vaccine based on clade 1 strain (A/Vietnam/1194/2004) could induce HI titer of at least 40 in 81–86% non-elderly adults and 76–79% elderly adults [113]. Moreover, two priming doses of MF59-adjuvanted H5N1 vaccine also induced significant neutralizing antibody titer against clade 2 strain (A/Turkey/Turkey/1/05) [113]. The cross-clade antibody responses significantly dropped 6 months later and yet could be boosted to above the protective levels by a single dose of the same vaccine [113]. In addition to adults, the MF59-adjuvanted H5N1 vaccine could also induce cross-clade antibody responses in pediatrics. Vesikari et al. found that two priming doses of MF59-adjuvanted H5N1 vaccine based on clade 1 strain (A/Vietnam/1194/2004) could induce significant microneutralization (MN) antibody titer against clade 2 strains (A/Indonesia/5/2005 and A/Anhui/1/2005) in pediatrics [114]. Despite significantly reduced antibody levels one year later, MN antibody titer against clade 2 strains could be boosted to more than 40 in most of the pediatrics by a single dose of the same vaccine [114]. These studies found that MF59 adjuvant was crucial to induce cross-clade antibody responses in homologous prime/boost immunizations. MF59 was also explored in heterologous prime/boost immunizations to induce cross-clade immunity. Galli et al. found MF59-adjuvanted clade 0-like H5N3 (A/duck/Singapore/1997) vaccine prime followed by MF59-adjuvanted clade 1 H5N1 (A/Vietnam/1194/2004) vaccine boost 6 years later could induce more rapid, higher, and longer duration of cross-reactive antibody responses against clade 0, 1, and 2 strains as compared to non-adjuvanted vaccine prime and MF59-adjuvanted clade 1 H5N1 vaccine boost [115]. This study indicated crucial roles of MF59 adjuvant in the H5N1 vaccine prime to induce cross-clade antibody responses after boost with heterologous H5N1 vaccine years later. The above studies support the incorporation of MF59 adjuvant in stockpiled H5N1 vaccines to facilitate the induction of cross-clade antibody responses following homologous and heterologous prime/boost immunizations.

In addition to the H5N1 vaccine, MF59-like SWE adjuvant was found to significantly increase the immunogenicity of the H7N9 vaccine, spare vaccine dose, and induce cross-protective immune responses against H7 viral strains of different lineages in ferrets [116]. Hatta et al. found that the MF59-like AddaVax adjuvant could significantly increase H7N9 vaccine efficacy and induce cross-protection against an antigenically distinct and highly pathogenic H7N9 viruses in ferrets [117].

#### 4.1.3. Safety

MF59-adjuvanted seasonal and pre-pandemic influenza vaccines were well tolerated in different age groups in humans [110,112,113,114,115]. No severe adverse events were related to MF59-adjuvanted influenza vaccination [110,112,113,114,115]. Yet, MF59-adjuvanted influenza vaccines were observed to induce more frequent local and systemic adverse reactions than non-adjuvanted vaccines [110,112,113,114,115]. The most common local adverse reaction was injection-site pain, and the most common systemic adverse reactions were myalgia and headache [112,113,115]. Most of the local and systemic adverse reactions were mild to moderate without the need for medical treatments.

#### 4.1.4. Mechanistic Insights

Khurana el al. looked into the cross-protective mechanisms of the MF59-adjuvanted H5N1 vaccine [118]. Whole-genome-fragment phage display libraries were used to map HA and NP epitopes capable of binding polyclonal antibodies of immune sera [118]. It was found that MF59-adjuvanted H5N1 vaccine induced a high frequency (60%) of anti-HA1 antibodies and broad anti-NA antibodies [118]. In contrast, non-adjuvanted and Alum-adjuvanted H5N1 vaccines induced a low frequency of anti-HA1 antibodies (23% and 32%, respectively) and rare anti-NA antibodies [118]. Moreover, MF59-adjuvanted H5N1 vaccine induced antibodies capable of binding long HA1 epitopes encompassing the receptor-binding domain (RBD), which is similar to those bound by broadly neutralizing antibodies [118]. In contrast, non-adjuvanted and Alum-adjuvanted vaccines mainly induced antibodies capable of binding short HA1 epitopes [118]. This study indicated that MF59 adjuvant could expand antibody repertoires to target protective HA sites and increase antibody avidities to properly folded HA1 [118]. In a different study [119], Khurana et al. found that MF59 adjuvant similarly enhanced influenza pandemic 2009 H1N1 vaccine-induced antibody diversity and affinity, more significantly expanded anti-HA1 than anti-HA2 antibodies, and induced antibodies capable of binding properly folded HA1 globular head. These studies provide important insights on how MF59 adjuvant broadens influenza vaccine-induced antibody responses against non-vaccine viral strains.

### 4.2. AS03

#### 4.2.1. Cross-Clade Protection against Pre-Pandemic H5N1 Vaccine

AS03 was also incorporated in the pre-pandemic H5N1 vaccine to spare vaccine doses [125]. Chu et al. found that two doses of AS03-adjuvanted H5N1 vaccine based on clade 1 strain (A/Vietnam/1194/2004) induced a 91.4% seroconversion rate against clade 2 strain (A/Indonesia/05/2005), while the non-adjuvanted vaccine only induced a 5.6% seroconversion rate against the same clade 2 strain [120]. Langley et al. found that two doses of AS03-adjuvanted H5N1 vaccine based on clade 2 strain (A/Indonesia/05/2005) induced significant neutralizing antibody titer against clade 1 strain (A/Vietnam/1194/2004) [121]. These studies support the incorporation of AS03 adjuvant in H5N1 vaccines to induce cross-clade antibody responses following a two-dose regimen.

AS03 adjuvant was also explored in prime/boost immunizations to induce cross-clade immunity. Schwarz et al. found that heterologous prime/boost immunization of AS03-adjuvanted H5N1 vaccine based on clade 1 (A/Vietnam/1194/2004) and clade 2 (A/Indonesia/05/2005) strains induced a 92.5% seroprotection rate against the clade 2 vaccine strain and a 98.1% seroprotection rate against the clade 1 vaccine strain [122]. This study also found that heterologous prime/boost immunization was superior to homologous prime/boost immunization to induce cross-clade immunity [122]. In support, homologous prime/boost immunization of the AS03-adjuvanted H5N1 vaccine based on the clade 1 strain (A/Vietnam/1194/2004) induced an 83.3% seroprotection rate against the clade 2 strain (A/Indonesia/05/2005) if the vaccines were given 6 months apart and a 41.5-54.5% seroprotection rate against the same clade 2 strain if the vaccines were given 21 days apart [122]. In another study, Leroux-Roels et al. found that two-dose priming of the AS03-adjuvanted H5N1 vaccine based on the clade 1 strain (A/Vietnam/1194/2004) and a single dose boost of AS03-adjuvanted H5N1 vaccine based on the clade 2 strain (A/Indonesia/5/2005) could induce rapid immune responses against the clade 2 vaccine strain [123]. In contrast, non-adjuvanted vaccine priming seemed to inhibit the induction of cross-clade immune responses [123]. Sun et al. found that homologous or heterologous prime/boost immunization of AS03-adjuvanted H5N1 vaccine based on the clade 1 (A/Vietnam/1203/2004) or clade 2 strain (A/Anhui/1/2005) could induce significant heterologous protection against antigenically distinct and highly pathogenic H5N2 viruses in ferrets [126].

#### 4.2.2. Safety

AS03-adjuvanted H5N1 vaccines were well tolerated in human subjects with no serious adverse events related to vaccination [120,121,122,123]. AS03-adjuvanted H5N1 vaccines also induced more frequent local and systemic adverse reactions than non-adjuvanted vaccines [120,121,122,123]. The most common local reaction was injection-site pain, and the most common systemic adverse reactions were myalgia and fatigue [120,121,122,123]. Most of the local and systemic adverse reactions were mild to moderate and self-resolved in days without the need for medical treatments [120,121,122,123]. It is also worth mentioning that the AS03-adjuvanted influenza pandemic 2009 H1N1 vaccine (Pandemrix) caused increased cases of narcolepsy in adolescents in Sweden and Finland 3-6 months after vaccination [127,128], while the non-adjuvanted vaccine failed to increase narcolepsy cases in the United States [129]. One study found that the HA peptide (275–287) of the pandemic virus could stimulate proliferation of hypocretin-responsive CD4^+^ T cells in narcolepsy patients [130]. Since the pathologic hallmark of narcolepsy is the loss of hypocretin neurons [130], immune responses against hypocretin might be responsible to the increased cases of narcolepsy following Pandemrix vaccination. Although MF59 and AS03 are both squalene emulsion-based adjuvants, the MF59-adjuvanted influenza pandemic 2009 H1N1 vaccine and seasonal influenza vaccine failed to increase narcolepsy cases [131,132]. Therefore, the unique combination of AS03 adjuvant and the pandemic influenza vaccine likely caused the increased cases of narcolepsy following Pandemrix vaccination.

#### 4.2.3. Mechanistic Insights

Khurana et al. used whole-genome-fragment phage display libraries to map antibody-binding regions of immune sera [125]. The AS03-adjuvanted H5N1 vaccine induced 10-fold higher anti-HA2 antibodies targeting the conserved H1/H5 region and also expanded antibody repertoires against the HA1 domain, especially against the long HA1 epitopes encompassing RBD [125]. The HA1 to HA2 ratio of bound clones was increased by 7-fold with the use of AS03 adjuvant, hinting the induction of anti-HA1 dominant antibody responses [125]. The AS03-adjuvanted H5N1 vaccine also induced anti-NA antibodies targeting the C-terminal region of NA, which is close to the sialic acid binding enzymatic site [125]. The AS03-adjuvanted H5N1 vaccine also increased antibody affinity against properly folded HA1 but not HA2 domains [125]. Moris et al. found that the AS03-adjuvanted H5N1 vaccine based on the clade 1 strain (A/Vietnam/1194/2004) could induce higher levels of long-lived memory B cells and cross-reactive and polyfunctional CD4^+^ T cells than the non-adjuvanted vaccine in young adults (18–60 years old) [124]. Due to the crucial roles of helper CD4^+^ T cells in stimulating antigen-specific B cell responses [133], cross-reactive and polyfunctional CD4^+^ T cells might contribute to the expanded antibody repertoires and the production of broadly cross-protective antibodies following the AS03-adjuvanted H5N1 vaccination [124].

### 4.3. TLR4 Agonists

MPL is a TLR4 agonist and has been a major component of several licensed adjuvants (e.g., AS01 and AS04) [93,154]. Glucopyranosyl lipid adjuvant (GLA) is a synthetic TLR4 agonist, and two major formulations of GLA have been explored as vaccine adjuvants: GLA-SE (stable emulsion) and GLA-AF (aqueous formulation) [135,136,155,156,157]. Studies found that TLR4 agonist-based adjuvants could assist influenza vaccines to induce cross-protective immunity. Pillet et al. found that GLA-SE could boost the plant-derived H5-VLP vaccine to induce protective HI titer in healthy adults [134]. Furthermore, the GLA-SE-adjuvanted H5-VLP vaccine induced significant polyfunctional and sustained heterologous CD4^+^ T cell responses against the influenza H2 protein [134]. Clegg et al. found that GLA-AF could boost the recombinant H5N1 hemagglutinin protein (rH5) based on the clade 1 strain to induce significant HI titer against heterologous clade 1 and clade 2 viruses and confer significant protection against clade 2 viral challenges in murine models [136]. GLA-AF also boosted rH5 based on the clade 2 strain to induce significant HI titer and protection against clade 1 strain in ferrets [136]. Although the majority of adjuvants have been licensed to boost IM vaccination, GLA-AF was explored to boost ID H5N1 vaccination. Carter et al. found that GLA-AF could assist the plant-produced H5-VLP vaccine to induce -higher HI titer against heterologous H5N1 viruses in murine models [135]. Furthermore, a single dose of the ID H5-VLP vaccine based on the clade 2 strain in the presence of GLA-AF conferred complete protection against lethal challenges of clade 1 strain in ferrets [135]. In human studies, the ID H5-VLP vaccine in the presence of GLA-AF induced more potent HI titer against heterologous clade 2 strain or clade 1 strain as compared to the IM H5-VLP vaccine in the presence of the same adjuvant or ID H5-VLP vaccine alone [135]. This study indicated that properly stimulated dermal APCs had a better ability than IM APCs to induce cross-protective immune responses [135]. Ko et al. found that a combination of MPL and CpG adjuvants could assist the influenza pandemic 2009 H1N1 vaccine to induce cross-protective immune responses against heterosubtypic H5N1 viruses in murine models [136]. Goff et al. found that a combination of synthetic TLR4 and TLR7 agonists could assist rHA or chimeric rHA to induce significant cross-protection against heterologous and heterosubtypic viruses in murine models [138]. Furthermore, the heterosubtypic protection was mainly due to the broadly cross-protective antibodies against HA stalks [138].

### 4.4. TLR7 Agonists

Imiquimod is a synthetic TLR7 agonist, and its topical 5% cream (Aldara) has been approved to treat genital warts, basal cell carcinoma, and actinic keratosis [158]. Topical imiquimod treatment (Aldara) followed by ID TIV was found to induce much higher seroconversion rates against all vaccine strains than ID TIV alone in young healthy adults [139]. Topical imiquimod followed by ID TIV also induced significant seroconversion against non-vaccine viral strains such as the antigenically drifted H3N2 strain [139]. 3M-052 was a synthesized TLR7 agonist containing an 18-carbon chain to limit its systemic distribution and induce more prominent local adjuvant effects [140]. Hoeven et al. found that one dose of rHA (H5N1, A/Vietnam/1203/04) in the presence of 3M-052 in stable emulsions induced 100% protection against homologous viral challenges in murine and ferret models, while rHA alone only induced partial protection [140]. Furthermore, rHA in the presence of 3M-052 in stable emulsions or liposomes induced significant neutralizing antibodies against clade 2 strains [140].

### 4.5. Flagellin

Flagellin is the major structural protein of bacterial flagellum and contains four domains (D0, D1, D2, and D3) [159]. D0/D1 domains are comprised of highly conserved N- and C-termini of flagellin [159]. D2/D3 domains are composed of highly variable internal region of flagellin [159]. Extracellular flagellin can be recognized by TLR5, and intracellular flagellin can be recognized by the cytosolic NLR family caspase recruitment domain (CARD)-containing protein 4 (NLRC4) inflammasome [160,161]. The D1 domain contributes to TLR5 binding [159,162,163], while the D0 domain contributes to NLRC4 inflammasome activation [164]. Foreign antigens can replace the highly variable D3 domain of flagellin or insert to its N or C-terminus without affecting its TLR5 activation ability [161]. Flagellin has been broadly explored as a highly immunogenic carrier for vaccine development against bacterial, viral, parasitic diseases, and cancer [161]. Wang et al. produced influenza PR8 HA-displayed M1 VLPs (HA/M1 VLPs) incorporating membrane-anchored flagellin (flagellin/HA/M1 VLPs) and found that the IM immunization of flagellin/HA/M1 VLPs could induce significant protection against heterosubtypic H3N2 viruses in murine models [141]. Due to the strong induction of respiratory epithelial cells with TLR5 expression to release cytokines and chemokines [161], flagellin has been also explored as a mucosal adjuvant [160]. Wang et al. found that IN immunization of flagellin/HA/M1 VLPs induced complete protection against heterosubtypic H3N2 viral challenges in murine models, while HA/M1 VLPs alone or in the presence of soluble flagellin showed little or no protection [142]. The above two studies indicated the importance of co-delivery of flagellin and HA/M1 VLPs in the induction of heterosubtypic immunity.

### 4.6. Saponin-containing Adjuvants

QS-21 is a highly purified saponin product from the bark extract of the South American tree, *Quillaja saponaria Molina* [165,166]. QS-21 has potent vaccine adjuvant effects if used alone at high doses [165]. Yet, a high-dose of QS-21 has the risk of inducing hemolysis in humans [165]. To improve its safety, QS-21 has been formulated with MPL in several adjuvant systems [93,167]. Other saponin-containing adjuvants have been also developed. Immunostimulating complexes (ISCOMs) are particulate antigen delivery systems comprised of vaccine antigens, phospholipids, cholesterol, and saponin [168,169]. ISCOMs are able to augment vaccine-induced humoral and cellular immune responses [168,170,171]. ISCOMs were explored to boost influenza vaccination and induce cross-protective immunity. Sambhara et al. found that ISCOMs could significantly increase H1N1 vaccine-induced HI titer and fully protect mice from heterosubtypic H2N2 viral challenges [143]. Further studies found that ISCOMs-induced heterosubtypic protection was correlated with CTL responses against a shared major histocompatibility complex (MHC) I epitope within HA of influenza H1 and H2 viruses [143]. ISCOM technology-based Matrix-M adjuvant was found to significantly increase trivalent virosomal vaccine-induced HI titer and protection against heterologous influenza type B but not type A viruses [144]. Matrix-M adjuvant also significantly increased HA nanoparticle-induced HI titer and protection against drifted H3N2 viruses in ferrets [145]. In another study, Cox et al. found that the Matrix-M-adjuvanted trivalent virosomal vaccine induced cross-protective immunity against highly pathogenic H5N1 and H7N7 viruses in mice and ferrets [146]. In addition to the seasonal influenza vaccine, the Matrix-M-adjuvanted virosomal H5N1 vaccine induced significant MN antibody titer against heterologous viruses in human subjects [147].

### 4.7. Enterotoxin Adjuvants

Cholera toxin (CT) of various strains of Vibrio cholerae, heat-labile enterotoxin (LT) of enterotoxigenic E. Coli, and their derivatives are the mostly studied mucosal adjuvants [172]. CT and LT belong to AB5 toxins composed of one A and five B subunits [172,173]. The A subunit catalyzes specific targets and is responsible for cell toxicity [172,173]. The B subunit forms a five-membered ring structure capable of binding to GM1-ganglioside receptors on target cells [172,173]. Quan et al. found that CT could significantly increase IN whole-inactivated PR8 (PR8i) vaccine-induced HI titer against heterologous and heterosubtypic viruses in murine models [148]. The CT-adjuvanted PR8i vaccine was found to confer complete protection against heterosubtypic H3N2 viruses, while the PR8i vaccine alone induced partial protection [148]. Furthermore, the heterosubtypic protection was mainly due to cross-reactive antibodies but not CD4^+^ or CD8^+^ T cells [148]. In an earlier study, Tamura et al. found that the CT B subunit (CTB) could significantly increase IN TIV-induced cross-protection against a drifted virus in murine models, while subcutaneous delivery of the same vaccine failed to induce similar levels of cross-protection [149]. An LT-adjuvanted influenza vaccine was approved for IN delivery in Switzerland (Nasalflu, Berna Biotech) during the 2000-2001 flu season [174]. However, it was pulled off the market due to the 19-fold increased risks of Bell’s palsy or facial paralysis in vaccine recipients [174]. Therefore, different LT mutants were developed to reduce the enterotoxicity of LT-based adjuvants, such as single-mutant LT (R192G) and double-mutant LT (R192G/L211A) [175,176]. Tumpey et al. found that the IN H3N2 vaccine in the presence of LT (R192G) adjuvant conferred complete protection against highly pathogenic H5N1 viruses in murine models, while the IN vaccine alone induced partial protection [150]. Furthermore, a subcutaneous vaccine in the presence of LT (R192G) or incomplete Freund’s adjuvant failed to confer protection against lethal H5N1 viral challenges [150]. B cells rather than CD8^+^ T cells were vital for the heterosubtypic protection [150].

### 4.8. CAF01

CAF01 is a two-component adjuvant comprised of cationic liposome dimethyl dioctadecyl ammonium bromide (DDA) and an immunomodulator trehalose 6,6′-dibeheneate (TDB) [177]. DDA is a quaternary ammonium compound comprised of one positively charged hydrophilic head and two hydrophobic 18-carbon tails and can self-assemble into liposome structures [178]. TDB is a glycolipid that is used to increase the stability and adjuvant effects of DDA liposomes [178]. Rosenkrands et al. found that the CAF01 adjuvant could significantly enhance TIV-induced humoral and cellular immune responses in BALB/c mice [151]. Furthermore, CAF01-adjuvanted TIV induced complete protection against heterologous H1N1 viral challenges, while TIV alone induced partial protection [151]. Christensen et al. found that CAF01 adjuvant significantly enhanced TIV-induced HI titer against homologous but not heterologous H1N1 viruses in ferrets [152]. Yet, CAF01-adjuvanted TIV more significantly reduced viral load and fever after heterologous H1N1 viral challenges as compared to TIV alone [152]. This study indicated that CAF01-adjuvanted TIV induced HI-independent cross-protective mechanisms in ferrets [152].

### 4.9. Physical Adjuvants

As an alternative to chemical adjuvants, physical adjuvants have been also explored to boost vaccination without introducing any chemicals or biological molecules into the body. Physical adjuvants utilize physical energies (e.g., laser, radiofrequency) to stimulate skin stress and alert innate immune systems to boost ID or transdermal vaccination. Due to the brief application of non-invasive or minimally invasive physical energies, physical adjuvants often induce minimal local or systemic adverse reactions and are less likely to induce long-term side effects. Laser adjuvants have been the mostly explored physical adjuvants so far and have been explored to boost influenza, nicotine, and cancer vaccination in preclinical animal models [179,180,181,182,183,184]. A near-infrared laser adjuvant was recently advanced to the clinical test and was found to stimulate significant cutaneous immune cell trafficking with good safety and tolerability in humans [185]. The intentional induction of microscopic photothermal damage by non-ablative fractional laser (NAFL) was found to safely boost ID influenza vaccination in murine models [186]. Interestingly, the NAFL significantly increased dissolving microneedle-based PR8i vaccine-induced immune responses and cross-protection against heterologous influenza H1N1 and H3N2 viruses in murine models [153]. We recently explored the use of radiofrequency energy to boost ID influenza vaccination and found that the radiofrequency adjuvant (RFA) could simultaneously augment influenza vaccine-induced humoral and cellular immune responses in murine models [187]. The RFA effects were comparable to MF59-like AddaVax adjuvant at 0.3 µg vaccine dose and superior to AddaVax adjuvant at 0.06 µg vaccine dose [187]. We recently found that the RFA-adjuvanted influenza pandemic 2009 H1N1 vaccine could induce heterologous protection against PR8 viruses, while the vaccine alone failed to induce heterologous protection in murine models (Li et al., manuscript in preparation).

## 5. Conclusions

Seasonal and pandemic influenza pose a huge threat to global public health. Current influenza vaccines mainly induce strain-specific protection. Better influenza vaccines are highly demanded to induce cross-protective immunity. In addition to the development of universal influenza vaccines, the incorporation of vaccine adjuvants into current influenza vaccines represents an attractive approach to induce cross-protective immunity. Squalene emulsion-based MF59 and AS03 adjuvants have been used to boost seasonal influenza vaccine efficacy in the elderly or spare pre-pandemic or pandemic influenza vaccine doses. MF59 and AS03 adjuvants have been found to also broaden influenza vaccine-induced immune responses against non-vaccine viral strains in human subjects. Different types of experimental adjuvants have been found to also assist current influenza vaccines to induce cross-protective immunity. Vaccine adjuvants hold a great promise to adjuvant current influenza vaccines to induce cross-protective immunity.

## Figures and Tables

**Table 1:** Influenza vaccine types.

Categories	Vaccine Types		
Number of viral strains	Trivalent (two type A, one type B)	Quadrivalent (two type A, two type B)	
Live-attenuated or inactivated	Inactivated (subunit, split-virion, virosome)	Live-attenuated	
Production method	Egg-based	Cell-based	Recombinant protein-based
Delivery routes (devices)	IM (traditional needles, needle-free Jet Injectors)	IN (Intranasal Sprayer)	ID (Intradermal Microinjection Systems, MicronJet600)
Usage	Seasonal	Pre-pandemic	Pandemic

**Table 2:** List of licensed vaccine adjuvants.

Adjuvants	Formulation and Type	Year of Approval	Vaccines
Alum	Aluminum salts	1930s	A number of vaccines (e.g., tetanus and diphtheria vaccines)
MF59	Squalene emulsion (Novartis)	1997	Seasonal influenza vaccine
AS04	MPL adsorbed on Alum adjuvant	2009	HPV vaccine
AS03	Squalene emulsion (GlaxoSmithKline)	2013	Pre-pandemic H5N1 vaccine
AS01	MPL/QS21 in liposome	2015	RTS,S malaria vaccine
CpG 1018	22-mer oligonucleotide	2017	Hepatitis B VLP vaccine

**Table 3:** Licensed adjuvants assist influenza vaccines to induce cross-protective immunity in human subjects.

Adjuvant	Vaccine and Immunization Regimen	Age Group	Cross-Protective Immunity	Safety	Reference
MF59	Trivalent influenza vaccine (TIV), subunit, single dose	Elderly	Heterologous HI GMT against seasonal influenza strains	More local and systemic adverse reactions. No serious adverse events related to vaccination	[110]
MF59	TIV, subunit, single dose	Elderly	Heterologous HI GMT against H3N2	Not studied	[111]
MF59	TIV, subunit, two doses (28 days apart)	Infants and young children	Heterologous HI GMT and seroconversion rates against all strains after the first and second dose	Higher rates of solicited adverse reactions. No serious adverse events related to vaccination	[112]
MF59	H5N1 vaccine (A/Vietnam/1194/2004, clade 1); subunit, two-dose prime (21 days apart) and one-dose boost (6 months later)	Non-elderly adults and elderly	HI GMT against clade 2 strain A/turkey/Turkey/1/05 detectable after two-dose priming and increased after boost	Common solicited adverse reactions, such as myalgia and headache. No serious adverse events related to vaccination	[113]
MF59	H5N1 vaccine (A/Vietnam/1194/2004, clade 1); two-dose prime (21 days apart) and one-dose boost (12 months later)	Pediatrics	HI GMT against clade 2 strains A/Indonesia/5/2005 and A/Anhui/1/2005 after prime and significantly enhanced after boost	Common mild–moderate local and systemic adverse reactions. No serious adverse events related to vaccination	[114]
MF59	H5N3 vaccine prime (A/duck/Singapore/97, clade 0); H5N1 vaccine boost (A/Vietnam/1194/ 2004, clade 1); two-dose prime (21 days apart) or three-dose prime with the third dose given 16 months later, two-dose boost 6 years later (21 days apart)	Non-elderly adults	Heterologous immunity against clade 0 strain A/Hong Kong/156/97, clade 1 strain A/Cambodia/R04050550/2007, and clade 2 strains A/Indonesia/5/2005, A/Turkey/15/2006, and A/Anhui/1/2005	Common mild–moderate local and systemic adverse reactions. No serious adverse events related to vaccination	[115]
AS03	Split-virion H5N1 vaccine (A/Vietnam/1194/2004, clade 1); two doses (21 days apart)	Non-elderly adults	HI GMT against clade 2 strain A/Indonesia/05/2005 after second dose	Higher rates of mild–moderate adverse reactions. No serious adverse events related to vaccination	[120]
AS03	Split-virion H5N1 vaccine (A/Indonesia/5/2005, clade 2); two doses (21 days apart)	Non-elderly adults	HI GMT against clade 1 strain A/Vietnam/1194/04 after second dose	More common adverse events. No serious adverse events related to vaccination	[121]
AS03	Split-virion H5N1 vaccine prime (A/Vietnam/1194/2004, clade 1); same strain or clade 2 strain boost (A/Indonesia/05/2005); One or two-dose prime (21 days apart) and one-dose boost 6 or 12 months later	Non-elderly adults	Seroconversion against clade 2 strain A/Indonesia/05/2005 after two-dose prime	No vaccine-related serious adverse events	[122]
AS03	Split-virion H5N1 vaccine prime (A/Vietnam/1194/2004, clade 1) and clade 2 strain boost (A/Indonesia/5/2005); two-dose prime (21 days apart); one or two-dose boost 14 months later (21 days apart)	Non-elderly adults	Rapid induction of HI GMT against clade 2 strain in adjuvanted vaccine-primed subjects	No vaccine-related serious adverse events	[123]
AS03	Split-virion H5N1 vaccine (A/Vietnam/1194/2004, clade 1); two doses (21 days apart)	Non-elderly adults	Cross-reactive CD4^+^ T cells against clade 2 strains A/Indonesia/5/2005 and A/Anhui/1/2005	Not studied	[124]

**Table 4:** Experimental adjuvants assist influenza vaccines to induce cross-protective immunity.

Adjuvant	Vaccine and Immunization Regimen	Study Subjects	Cross-Protective Immunity	Safety	Reference
SWE	Split virion vaccine of A/Shanghai/2/2013 (H7N9)-A/Puerto Rico/8/34 (PR8)-IDCDC-RG32A.3; two doses (21 days apart); IM	Ferrets	HI titer against heterologous Eurasian and North American Lineages of H7N2, H7N3, H7N7, and H7N9 viruses, Heterologous protection against A/Anhui/1/2013 (H7N9) virus	Not studied	[116]
AddaVax	Inactivated whole virus vaccine of reassortant A/Hong Kong/125/2017 (H7N9) and PR8 backbone; two doses (28 days apart); IM	Ferrets	HI titer and protection against highly pathogenic heterologous H7N9 rGD/3-NA294R virus	Not studied	[117]
GLA-SE	Plant-produced H5-VLP (A/Indonesia/05/2005, clade 2); two doses (21 days apart); IM	Non-elderly adults	Polyfunctional and sustained heterologous CD4^+^ T cell responses against influenza H2 protein	Higher incidences of solicited symptoms and mostly mild or moderate	[134]
GLA-AF	Plant-produced H5-VLP (A/Indonesia/05/2005, clade 2); two doses (21 days apart); ID	Non-elderly adults	HI GMI and seroconversion against heterologous clade 2 virus A/Anhui/1/2005 and HI GMI and seroconversion against clade 1 virus A/Vietnam/1203/2004	Transient erythema; no serious adverse events related to vaccination	[135]
GLA-AF	Plant-produced H5-VLP (A/Indonesia/5/05, clade 2); two doses (21 days apart) in mice and guinea pigs; one dose in ferrets; IM or ID	Mice, guinea pig, and ferrets	HI titer and protection against heterologous virus A/Duck/Hunan/795/2002 in mice and protection against clade 1 strain A/Vietnam/1203/04 in ferrets	Guinea pigs: no temperature spikes or body weight decrease; Ferrets: no local reactions or body temperature increase	[135]
GLA-AF	Recombinant HA (rHA) of H5N1 A/Vietnam/1203/04 or A/Indonesia /05/05 viruses; one dose in mice and one or two doses (21 days apart) in ferrets; IM	Mice and ferrets	Cross-clade HI titer and protection	Not studies	[136]
MPL and CPG combination	Split virion vaccine of A/California/04/2009 H1N1; one or two doses (21 days apart); IM	Mice	HI titer and protection against heterosubtypic reassortant H5N1 A/Vietnam/1203/2004 virus	Not studied	[137]
TLR4 and TLR7 agonist combination	rHA of PR8 H1N1; chimeric rHA (same stalk region); one dose (rHA) or 3 doses (chimeric rHA); IM	Mice	Heterologous protection against A/California/04/2009 H1N1 virus; Heterologous protection against B/Florida/04/2006 (Yamagata lineage); Heterosubtypic protection against reassortant A/Vietnam/1203/2004 H5N1 virus	Not studied	[138]
Imiquimod (Aldara)	TIV (Intanza); one dose; ID	Healthy student volunteers	Heterologous HI and MN titer against non-vaccine strains	More common redness and swelling; no serious adverse events related to vaccination	[139]
3M-052	rHA of A/Vietnam/1203/04 H5N1; two doses with 28 days apart in mice; one dose in ferrets; IM	Mice and ferrets	Cross-clade antibody binding in mice and heterologous protection against A/Whooper Swan/Mongolia/ 244/05 H5N1 virus in ferrets	Not studied	[140]
Membrane-anchored flagellin	HA/M1VLPs based on PR8 virus; two doses (28 days apart); IM or IN	Mice	Heterosubtypic protection against A/Philippines/82 (H3N2) virus	Not studied	[141,142]
ISCOMs	Fluzone^®^ monovalent subunit vaccine of A/Taiwan/1/86 H1N1; two doses (21 days apart); subcutaneous	Mice	Heterosubtypic protection against A/Japan/305/57 (H2N2) virus due to CTL but not antibody responses	Not studied	[143]
Matrix-M	Trivalent virosomal vaccine; one to three doses (21 days apart); IM	Mice	Heterologous protection against influenza A and B strains	Not studied	[144]
Matrix-M	TIV; two doses (21 days apart); IM	Mice and ferrets	Heterologous protection against H3N2 strain A/Texas/71/2007; Heterosubtypic protection against H5N1 and H7N7 viruses	Not studied	[145,146]
Matrix-M	Inactivated virosomal H5N1 vaccine of A/Vietnam/1194/2004 (clade 1); two doses (21 days apart); IM	Non-elderly adults	HI titer against heterologous clade 1 virus (A/Cambodia/R0405050/2007) and cross-clade HI titer against clade 2 viruses (A/Indonesia/5/2005 and A/turkey/Turkey/1/2005)	Not studied	[147]
Cholera toxin	Inactivated PR8 vaccine; two doses (14 days apart); IN	Mice	Heterologous protection against H1N1 and heterosubtypic protection against H3N2 virus (A/Philippines/82)	Not studied	[148]
Cholera toxin B subunit	HA of A/Fukuoka /C29/85 (H3N2) or A/Sichuan/2/87 (H3N2); one dose; IN	Mice	Heterologous protection against H3N2 virus (A/Guizhou/54/89-X)	Not studied	[149]
LT (R192G)	Whole-inactivated X-31 (H3N2) vaccine; three doses (7 days apart); IN	Mice	Heterosubtypic protection against H5N1 virus	Not studied	[150]
CAF01	TIV; two doses (3-4 weeks apart); subcutaneous	Mice and ferrets	Heterologous protection against H1N1 viruses	Not studied	[151,152]
NAFL	Inactivated PR8 vaccine; one dose; transdermal	Mice and miniature pigs	Heterologous and heterosubtypic protection against H1N1 and H3N2 viruses in mice	Minimal local reactions in miniature pigs	[153]

## Data Availability

Not applicable.
